# Decisional needs and interventions for young women considering contraceptive options: an umbrella review

**DOI:** 10.1186/s12905-024-03172-2

**Published:** 2024-06-08

**Authors:** Marit Müller De Bortoli, Sienna Kantymir, Lissa Pacheco-Brousseau, Bente Dahl, Elisabeth Holm Hansen, Krystina B. Lewis, Qian Zhang, Victoria Cole, Thomas Westergren, Dawn Stacey

**Affiliations:** 1 Department of Nursing and Health Sciences, Faculty of Health and Social Sciences, University of South - Eastern Norway , Kjølnes ring 56, Porsgrunn, N3918 Norway; 2 Ottawa Hospital, Ottawa, Canada; 3 School of Rehabilitation Sciences, University of Ottawa, Ottawa, Canada; 4 University of South - Eastern Norway, Bakkenteigen, Norway; 5 University of South - Eastern Norway, Public health nurse, Porsgrunn, Norway; 6 School of Nursing, University of Ottawa, University of Ottawa Heart Institute, Ottawa, Canada; 7 School of Nursing, University of Ottawa, Ottawa, Canada; 8 Research Librarian, University of Ottawa, Ottawa, Canada; 9 University of Agder & University of Stavanger, Kristiansand & Stavanger, Norway; 10 School of Nursing, Centre for Implementation Research Ottawa Hospital Research Institute, Ottawa Hospital Research Institute, Ottawa, Canada

**Keywords:** Contraception, Decisional needs, Decision making, Decision support intervention, Young women, Patient decision aids

## Abstract

**Background:**

Although women face a wide range of contraceptive options, globally, young women are at risk of unintended pregnancies. Our umbrella review aimed to determine the decisional needs of nulligravida women aged 11 to 30 considering contraceptive options and identify effective interventions to support their involvement in making decisions about contraceptive use.

**Methods:**

We followed Joanna Briggs Institute methods for umbrella reviews, theoretically guided by the Ottawa Decision Support Framework. We searched six electronic databases. Two reviewers independently screened citations, extracted data, and appraised quality using AMSTAR2. We analysed findings descriptively.

**Results:**

Of 124 citations, we identified 11 reviews of variable quality (critically low to moderate quality): Six reported decisional needs and 5 reported on interventions. Decisional needs of young women were: (a) information needs about contraceptive options (e.g., mechanism of actions, eligibility, administration, side effects); (b) unclear values (concerns about hormone use) and features of different options (based on their religious values); and (c) need for support and resources (support from society and need for privacy). Compared to controls, decision support interventions including patient decision aids and patient education material increased knowledge and improved discussion of options with their clinicians.

**Conclusion:**

Young women making contraceptive decisions experience unmet decisional needs. Effective interventions such as patient decision aids and general patient education materials may address their decisional needs and enhance their level of participation in making contraception decisions. Implications and contribution to the field: Young women’s decisional needs when considering contraceptive use are informational needs, unclear values (including religious influences), need for support and resources when facing this decision. Interventions, such as patient decision aid and patient education material can, address decisional needs by improving young women’s knowledge about contraceptive options.

**Supplementary Information:**

The online version contains supplementary material available at 10.1186/s12905-024-03172-2.

## Introduction

Despite availability of numerous contraceptive options [1], there continues to be unintended pregnancies in young women. Younger women who become pregnant are at higher risk of mortality and morbidity [2,3], and are more likely to have social and economic consequences including dropping out of school or challenges with job stability [2].

According to the United Nations and World Health Organization (WHO), family planning with contraceptive counselling is a human right for all women and is pivotal for obtaining gender equality, decreasing poverty, and enhancing women’s bodily autonomy [1,4]. Young women are eligible for the same contraceptive options as older women [1]. However, only 60% of young women aged 15–19 receive modern contraceptives options compared to 75% of women over 30 years (2020) [5]. The decision about which contraceptive method to select presents unique challenges for young nulligravida women [6,7]. Common side effects of contraceptives include pain on insertion, changes in menstrual bleeding, deep vein thrombosis, acne, and headaches; some side effects may be considered positive such as less bleeding or reduced hormonally mediated premenstrual symptoms (e.g. headaches, bloating) [8].

Young women are more likely to have their decisions influenced by their changing cognitive abilities, emotions, risk taking behaviours, and physical characteristics [9]. Specific barriers limiting access to contraceptive options include lack of information about, cultural and societal stigmas, attitudes of healthcare professionals, and legal issues can limit access to contraceptives [2]. Other social factors such as gender, income, education, and inequality may also inhibit their access to contraceptive services [2]. Furthermore, misperceptions and myths about contraceptive options are common (e.g. need for a pelvic examination, concerns for future infertility, weight gain, cancer risk, among others) [7] interfere with young women using contraceptives and results in higher chance of unintended pregnancies [7,10].

There are international efforts to increase the use of modern, effective, and safe long-acting reversible contraceptives (LARC) such as intrauterine devices (IUD) and contraceptive implants. Yet, LARC may be more painful during the insertion especially for nulligravida women and potential risks that should be considered during the decision making process [11].

It is paramount to respect young women´s reproductive rights and avoid providing biased information or using coercion towards using specific types of contraceptives [11]. Engaging young women in making contraceptive decisions can enhance their understanding of the benefits and risk/harm (including side effects), identify potential barriers to accessing contraceptives, and augment their feeling of autonomy [12]. Thereby, supporting young women to select an option that is congruent with their informed values for features and outcomes of options. To support them in achieving this quality decisions, it is essential to understand their decisional needs and determine if effective decision support interventions are available to address their needs [13]. This includes their understanding of the benefits and risks/harms (including side effects), identifying potential barriers to accessing contraceptives, and selecting an option that is congruent with their own values for features and outcomes of options [12]. To achieve a quality decision, decisional needs can be addressed with tailored decision support interventions such as patient decision aids and decision coaching [13].

Although recent umbrella reviews investigated components of decisional needs and identify interventions to support decision making about contraceptives [14,15], none have focused on the perspectives of young women making this decision [2,13]. The overall aim of our umbrella review was to summarize the evidence specific the decisional needs and effective decision support interventions for young women (aged 11–30) considering contraceptive options.

### Review questions

1. *What are the decisional needs of young women facing contraceptive options?*
2. *What are the effects of interventions that support young women in making decisions about contraceptive options on their knowledge, participation in decision making and experiences?*

## Methods

We conducted an umbrella review following the Joanna Briggs Institute (JBI) methodology for umbrella reviews [16] and theoretically informed by the Ottawa Decision Support Framework (ODSF) [13]. The protocol was registered in Prospero 2023 CRD42023402147. We reported the findings using the Preferred Reporting Items for Systematic Reviews and Meta-Analyses (PRISMA) guidelines [17].

According to the ODSF [13,18], there are three key elements to consider for supporting people to be involved in decision making: decisional needs, decision support interventions and decisional outcomes. Common decisional needs include decisional conflict, inadequate knowledge, unrealistic expectations, unclear values, inadequate support and resources, and clinical and personal needs [19]. Decision support interventions that can be used to address the decisional needs include clinical counselling, patient decision aids, or decision coaching [13]. Decisional outcomes are focused on enhancing the quality of the decision and quality of the decision making process, ultimately indicative of a reduction in decisional needs.

### Eligibility criteria

The inclusion and exclusion criteria are described using JBI framework: Population, Intervention and phenomenon of Interest, Context, Outcomes of Interest, and Study design (PICOS) [16,20].

#### Population

We included reviews of young women after menarche and no previous pregnancy (i.e., approximately 11 to 30 years old). For reviews of women of all age groups considering contraception, we only included those who reported specific findings for young women (≤ 30 years). For reviews that included nulliparous and parous women, we included reviews if more than 50% of participants did not report being or ever having been pregnant. We excluded reviews that investigated the perspectives of males, significant others, family members, or healthcare professionals. We also excluded reviews of women postpartum, seeking abortion, or with a specific health condition (e.g., rheumatoid arthritis, cancer, psychiatric disorders).

#### Intervention and phenomena of interest

The phenomena of interest included decisional needs about contraception decision making (research question 1) and interventions to support decision making about contraceptive options (research questions 2). Interventions for example included patient decision aids or patient education materials. Reviews were excluded if they focused on emergency contraception, pregnancy decisions, abortion, human immunodeficiency virus (HIV) or sexually transmitted diseases (STD) prevention, actual use of contraceptives (e.g., prevalence studies), or sterilization.

#### Context

Eligible reviews for decisional needs were conducted in global north countries as indicated in the World Bank’s interactive map [21]. Reviews were excluded if they were conducted in global south countries given the differences in healthcare services supporting young women considering contraception (e.g., availability of contraceptives, cost, cultural practices) [22,23].

Eligible reviews for interventions to support decision making were from any country. That interventions may be universally applied across higher and lower income countries and decision support interventions may be adapted for use in other countries.

#### Outcomes of interest

Eligible reviews reported on any decisional needs (e.g., decisional conflict, inadequate knowledge, unrealistic expectations, unsupported, unclear values, clinical and personal needs and/or other outcomes indicating decisional needs).

Outcomes for interventions to support decision making included improved knowledge, participation in decision making, and their experiences (e.g., decisional conflict, satisfaction). Reviews were excluded if they exclusively reported uptake or use of options.

#### Study design

Eligible studies included any peer-reviewed knowledge synthesis studies including systematic reviews with or without meta-analysis, scoping reviews, qualitative systematic reviews, realist reviews, and rapid reviews [24]. Studies were excluded if they were individual studies, not peer-reviewed, brief reports, editorials, commentaries, protocols, conference abstracts, literature reviews, narrative reviews, dissertations, or theses.

### Information sources

The search strategy was developed with a Research Librarian (VC) and peer reviewed by another information specialist (ND) using the PRESS guideline [25]. The search was conducted in six electronic databases: MEDLINE (OvidSP) (see supplement), Embase (OvidSP), PsycInfo (OvidSP), CINAHL(EBSCOHost), Cochrane Database of Systematic Reviews (CDSR) and Web of Science (Core Collection) from January 2000 to March 2023. The time limit of 2000 reflects the United States Food and Drug Administration approval of the IUD Mirena® (Berlex Laboratories, Wayne, NJ); the first levonorgestrel-releasing intrauterine system to be approved for use [26]. No language limits were applied to the search. To capture the breadth of research on this topic, we searched the following concepts using a combination of subject headings and keywords: contraception, decision making and childbearing populations. In drafting the search strategy, the concept of “contraception” was informed by Mack et al.’s Cochrane review [27]. Canada’s Drug and Health Technology Agency’s systematic review search filter was adapted to include scoping, rapid and realist reviews and used for searches in the electronic data bases [28,29].

### Selection process

Using Covidence (Veritas Health Information, Melbourne, Australia, 2023), two independent reviewers (LP, MMDB) used a two-level screening process: (a) titles and abstracts; and (b) full text articles. All discrepancies were resolved through discussion and when unsure, a third reviewer was involved (DS). For identified umbrella reviews, included reviews were screened using the two-step process by two independent reviewers (MMDB, DS).

### Data collection process

Two independent reviewers (MMDB, DS, SK, QZ) extracted the data using an electronic standardized data extraction form. The extraction form followed the JBI Data Extraction Form for Review for Systematic Reviews and Research Syntheses (e.g., authors, year, study design etc.) [16] and included relevant ODSF elements, specifically decisional needs and decision support interventions) [13,18]. If inadequate details were reported in the systematic reviews on the decision support interventions, we consulted the primary study to extract this additional data. We reviewed extracted data to determine whether the decision support interventions met qualifying criteria to be a patient decision aid according to the International Patient Decision Aid Standards [30,31]. If the intervention did not fulfil these criteria it was defined as patient education material.

### Quality and risk of bias assessment

At least two review authors (MMDB, QZ, DS, TW) independently assessed the quality of included systematic reviews using the AMSTAR2 [32] and two additional items from the JBI Critical Appraisal Checklist for Systematic Reviews and Research Syntheses (e.g., recommendations for policy, directions for new research [33] (see supplement).

To determine confidence in the results from each included review, we relied on AMSTAR2 critical items 4, 7, 9, 11, 13, and 15 [32]. The other AMSTAR2 items were considered non-critical [32]. Results were then rated as follows: 4) critically low confidence if there were more than one critical flaw 3) low confidence if there was one critical flaw 2) moderate confidence if there was more than one non-critical weaknesses; and 1) high confidence if there was one or fewer non-critical weaknesses [32].

For the primary studies of decision support interventions, we used the risk of bias results as reported in the systematic review.

## Synthesis methods

Decisional needs were deductively analysed using the coding manual for the ODSF [13,18]. The interventions were analysed according to JBI summary of evidence for umbrella reviews [16]. Results were reported with their confidence rating as described above [32]. Findings were descriptively reported and summarized in tables.

### Patient and public involvement

We included, as part of the author group, three women in the target age group (< 30 years of age) to ensure the experiences of young women faced with these decisions were captured in our study design, conduct, and dissemination. They contributed to every step of the umbrella review conduct, from conceptualization to writing the manuscript.

## Results

### Characteristics of included reviews

**Fig. 1:**
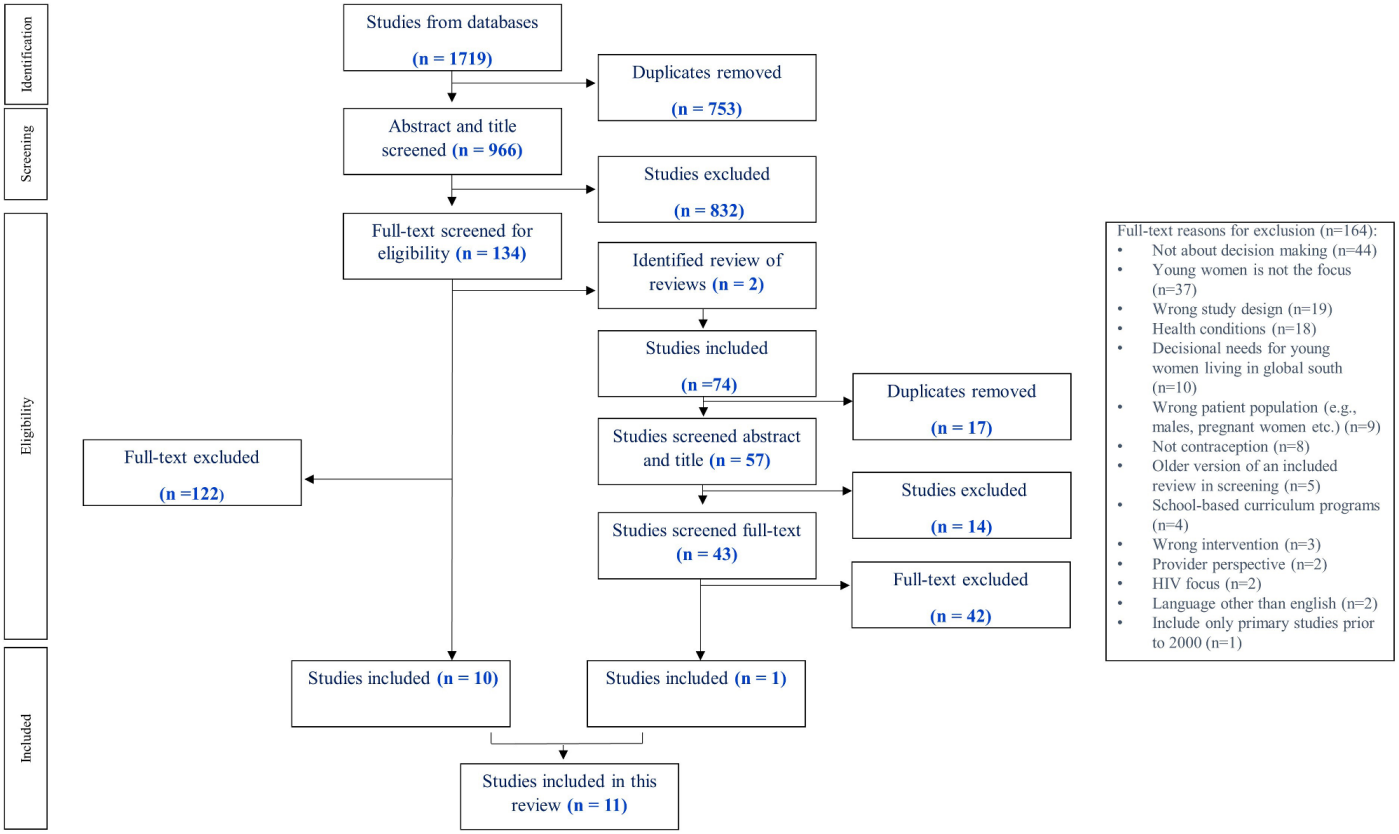
Preferred Reporting Items for Systematic Reviews and Meta-Analyses flow diagram

The database search identified 1719 records. After duplicates were removed, 966 records were screened resulting in 11 included reviews (see Fig. 1). The included reviews were conducted in the USA (*n* = 5), United Kingdom (*n* = 3), Canada (*n* = 1), Brazil (*n* = 1), and a multinational (UK, Belgium, United States of America, Switzerland) (*n* = 1). Most common reasons for excluding reviews at full text screening were: not focused on young women, not about decision making, limited to global south (decisional needs only), healthcare professional perspectives, male participants, post-partum (> 50% of sample), parent perspective, women undergoing abortion, women utilizing emergency contraception (see supplement S1). Some reviews reported on the same primary studies (see Table 1 and supplementS1).

**Table 1:** Characteristics of the included reviews for decisional needs (N = 6) and decision support interventions (N = 5)

Study details	Review aim	Type of review with number of studies	Number of databases (years)	Population age range	# primary studies eligible (population)
Decisional needs					
Baxter (2011) UK [34]	Examine young women and health care professionals’ views on contraceptive services	Systematic review of 59 studies	12 databases (1995–2008)	15–25	4 studies (N = 243)
Daley (2014) USA [38]	Synthesize research on contraceptive decision-making among adolescents	Meta-ethnography with 14 studies	8 databases (2000–2012)	12–21	8 studies (N = 255)*
Fox (2018) USA [42]	Describe clients’ preferences regarding contraceptive counselling approaches in the family planning setting	Systematic review of 26 studies	16 databases (1992–2011)	14–29	9 studies (N = 177)
Kirubarajan (2022) Canada [39]	Determine awareness of LARCs in young people and characterize knowledge gaps, predictors of knowledge, information sources of adolescents	Systematic review and meta-analysis of 40 studies	3 databases (2001–2021)	12–25	32 studies (N = 8348)
Reis (2018) Brazil [36]	Review studies concerning the decision-making process of adolescents who use LARCs	Systematic review of 9 studies	8 databases (prior to May 2018)	12–19	7 studies (N = 3741)
Ti (2022) USA [37]	Describe values and preferences of adolescents and young adults related to contraception	Systematic review of 55 studies	10 databases (2005–2020)	10–25	36 studies (N = 4575)
**Decision support interventions**					
Blank (2012) UK [43]	Explore effectiveness of services which provide contraception to young people delivered in developed countries	Systematic review and narrative synthesis of 23 studies	11 databases (1995–2008)	< 25	1 study (N = 949)
Cavallaro (2019) UK, Belgium, USA, Sw [41]	Investigate comparative effectiveness of contraceptive counselling strategies on contraceptive behaviour and satisfaction	Systematic review of 61 studies	6 databases (1990–2018)	11–29	2 studies (N = 1156)
Goueth (2022) USA [42]	Effectiveness of technology-based contraceptive decision aids	Systematic review and meta-analysis of 18 studies	7 databases (2005–2022)	12–30	7 studies (N = 3612)
Jones (2022) USA [40]	Explore how effective are currently available contraceptive decision aids	Systematic review of 10 studies	2 databases (2011–2021)	11–30	4 (N = 2886)**
Walker (2020) UK [44]	Resources for healthcare professionals to guide or structure the process of conducting an integrated sexual and reproductive health consultation	Scoping review of 17 studies	8 databases (1998–2017)	18–29	1 study (N = 21)

LARC= Long-Acting Reversible Contraception; Sw= Switzerland; UK= United Kingdom; USA=United States of America *The authors refer to Noblit, G. W., & Hare, R. D. (1988). Meta-ethnography: Synthesizing qualitative studies. Newbury Park, CA: Sage Publications **The authors state that due to heterogeneity of outcomes and methods in primary studies, the authors has performed a qualitative synthesis

### Young women’s decisional needs

Six reviews reported on decisional needs, of which five were qualitative systematic reviews [34–37] including a meta-ethnography [38], and one systematic review combined a meta-analysis with qualitative data [39] (see Table 2). The evidence on decisional needs of young women considering contraception options were based on two low [35,39], and four critical low [34,36–38] reviews (see Table 3). According to JBI critical appraisal checklist, only two reviews [37,39] included information on recommendations for policy, and four [34,35,37,39], proposed areas for future research (see supplement). The four broad decisional needs identified were informational, values, support and resources, and personal characteristics (see Table 4).

**Table 2:** Quality rating of included reviews based on AMSTAR 2

First author (year)	Item 1 PICO	Item 2 Protocol	Item 3 Selection of study design	Item 4 Compre-hensive search	Item 5 Duplicate study selection	Item 6 Extract in duplicate	Item 7 Justify exclusion	Item 8 Detailed included studies	Item 9 Risk of bias (RoB) assessed	Item 10 Sources of funding	Item 11 Meta-analysis performed	Item 12 Meta-analysis; potential RoB	Item 13 Account for RoB in discussion	Item 14 Hetero-geneity	Item 15 Publica-tion bias	Item 16 Conflict of interest	Overall rating	
Decisional needs																		
Baxter (2011)	No	No	No	Yes	No	No	No	Yes	Yes	No	N/A	N/A	No	No	N/A	Yes	Critical low	
Daley (2014)	Yes	No	Yes	Yes	No	No	No	Yes	No	No	N/A	N/A	No	No	N/A	Yes	Critical low	
Fox (2018)	No	No	No	Partial yes	Yes	Yes	No	Yes	Yes	Yes	N/A	N/A	Yes	No	N/A	Yes	Low	
Kirubarajan (2022)	Yes	No	Yes	Yes	Yes	Yes	No	No	Yes	No	Yes	Yes	Yes	Yes	Yes	Yes	Low	
Reis (2018)	Yes	No	Yes	Yes	Yes	Yes	No	Partial Yes	No	No	N/A	N/A	No	No	N/A	Yes	Critical low	
Ti (2022)	Yes	No	Yes	Yes	Yes	Yes	No	Yes	No	No	N/A	N/A	Yes	Yes	N/A	Yes	Critical low	
**Decision support interventions**																		
Blank (2012)	Yes	No	Yes	Partial Yes	No	No	No	Yes	Yes	No	N/A	N/A	Yes	No	N/A	Yes	Critical low	
Cavallaro (2020)	Yes	Yes	No	Yes	No	No	No	No	No	No	N/A	N/A	No	No	N/A	Yes	Critical low	
Goueth (2022)	Yes	Yes	Yes	Partial Yes	Yes	Yes	No	Yes	Yes	No	Yes	Yes	Yes	Yes	Yes	Yes	Low	
Jones (2022)	Yes	No	Yes	Yes	Yes	Yes	Partial yes	Yes	Yes	No	N/A	N/A	Yes	Yes	N/A	Yes	Moderate	
Walker (2020)	No	No	No	Yes	Yes	No	No	Partial Yes	No	No	N/A	N/A	No	No	N/A	Yes	Critical low	

N/A= Not applicable

**Table 3:** Decisional needs of young women considering contraception options (n=6 reviews)

Review first author (year)	Countries of included primary studies	Data collection method(s) (n)	Information needs	Values	Support and resources	Personal characteristics	Confidence in results†
Baxter (2011) ^[[34]]^	UK(n = 4)	Interview (n = 56), survey (n = 167), interview and survey (n = 20)		Yes	Yes	Yes	Critical low
Daley (2014) ^[[38]]^	USA (n = 8)	Interviews (n = 148), focus groups (n = 102), grounded theory (n = 5)	Yes	Yes	Yes	Yes	Critical low
Fox (2018) ^[[35]]^	USA (n = 8), Belgium (n = 1)	Interviews (n = 100), focus group (n = 26), interview and survey (n = 35), interview and focus group (n = 16)	Yes				Low
Kirubarajan (2022) †† ^[[39]]^	USA (n = 25) UK (n = 2), Norway (n = 1), Netherlands (n = 1), Australia (n = 2), New Zealand (n = 1)	Interviews (n = 310), focus group (n = 88), survey (n = 7340), interviews and survey (n = 14), focus group and survey (n = 596)	Yes				Low
Reis (2018) †† ^[[36]]^	USA (n = 6), Greece (n = 1)	Cohort (n = 3441), survey (n = 300)	Yes			Yes	Critical low
Ti (2022) ^[[37]]^	USA (n = 25), UK (n = 7), Canada (n = 1), Puerto Rico (n = 1), Australia (n = 2), Austria (n = 1), New Zealand (n = 1)	Interviews (n = 563), focus group (n = 15), survey (n = 3054), cohort (n = 731), case series (n = 20), RCT (n = 130), focus group and interviews (n = 62)	Yes	Yes	Yes		Critical low

† AMSTAR2 total score= Measurement Tool to Assess systematic Reviews; UK= United Kingdom; USA=United States of America † † Exclusively about LARC; blank=not reported

**Table 4:** Effects of interventions to support decision making process for young women in regards of contraception

**First author (year) of primary study study; study design; (sample size); country**	Review first author (year) Risk of bias of primary study reported in review‡	**Intervention** Number of contraceptive methods included	Comparator	Results		
				Knowledge	Participation in decision making	Experiences
Chewning (1999) Longitudinal (N = 949) USA ^[[48]]^	Cavallaro (2019) Risk of bias not reported Blank (2012) Good quality	Contraceptive decision making programme, followed with clinic visit Control group (standard patient education and clinic visit) No details given	NR	Compared to control: Increased knowledge **(**p ≤ 0.000)	NR	Compared to control: more confidence in oral contraceptive efficacy (p ≤ 0.000)
Antonishak (2015) RCT (N = 2284) USA ^[[50]]^	Jones (2022) Medium risk of bias	Online birth control support network; website Bedsider.org 17 methods	Control group (no exposure)	Compared to control: more familiar with different methods (p = .00); no difference in knowledge of relative effectiveness.	NR	NR
Marshall (2017) Qualitative interviews (N = 21) USA ^[[53]]^	Walker (2020) Risk of bias not reported	Contraceptive decision support tool plus; website, LARC videos and counselling (My control navigator) “Birth Control Navigator” 16 methods	No comparator	Informative; missed some information (e.g. sexual pleasure); narrowed down options; less biased information	NR	User experienced it to be useful before consultation
Dehlendorf (2019) cRCT (N = 748 under 30 years) (73.2% of total participants aged 15–29) USA ^[[45]]^	Goueth (2022) Good overall rating	Contraceptive decision support tool; website (“My Birth Control”) https://clinic.mybirthcontrol.org/ 11 methods	Control group (standard counseling)	Compared to control: Increased knowledge; Answered question correctly on IUDs (OR, 2.47; CI, 1.75–3.49; p = < .01)	Compared to control: no difference on making the decision themselves (> 72% both groups)	Compared to control: no difference in overall decisional conflict 25.4% vs 22.6% (p = .41)
**Patient education materials**						
Gilliam (2014) Pilot RCT (N = 52) USA ^[[51]]^	Jones (2022) Medium risk of bias Goueth (2022) Fair overall rating	Waiting-room application followed by standard counselling (provided with same day contraceptives) Not specified, but emphasized LARC (15 minute)	Control group (standard contraceptive counselling care)	Compared to control: Increased knowledge; (p = .0001)	NR	Intervention group responded they would recommend app to a friend
Mesheriakova, (2017) Prospective cohort study (N = 120) USA ^[[54]]^	Goueth, 2022 Risk of bias not reported	Interactive, individually tailored application (“Health-E You”) Not specified	No comparator	Increased knowledge (p ≤ .001)	NR	NR
Hebert (2018) RCT (N = 207) USA ^[[55]]^	Cavallaro (2019) Risk of bias not reported Jones (2022) High risk of bias Goueth (2022) Fair overall rating	Waiting-room application with LARC videos and counselling (“miPlan”) All method showing tiered effectiveness; focus on LARC (10 minutes)	Control group (standard clinic visit)	Compared to control Increased knowledge (p < .001)	NR	NR
Sridhar (2019) Pre-post test (N = 120) USA ^[[49]]^	Jones, 2022 Low risk of bias	Comics paper format (www.birthcontroltales.com) Injection, IUD, implant, combined hormonal	No comparator	Increased perceived knowledge (p = .001); 80% easy to understand	NR	75% appreciated
Tebb (2021) cRCT (18 sites, N = 1360 Latina/Hispanic girls) USA ^[[52]]^	Goueth, 2022 Fair overall rating	Interactive, individually tailored application (“Health-E You/Salud iTu”) with tailored recommendations based on preference; printout for use in counselling with provider Not specified	Control group (baseline survey on ipad, followed by standard counselling)	Compared to control: Increased knowledge 3.3 (± 1.6) at baseline to 4.6 (± 1.7) after app use (p < 0.001); 43% too much information	Compared to control: higher OR of discussing method with provider, (OR 2.22 (0.98, 5.01)); 70% intervention arm felt it helped quality of the health visit	High satisfaction with intervention
Manlove (2020) **Manlove (2021)** RCT (N = 1124) Replication study (N = 871) USA ^[[46][47]]^	Goueth, 2022 Good overall rating	Reproductive health application (“Pulse”) to provide sexual and reproductive health content for young Black and Latinx women Not specified	Control group (access to general health application)	Compared to control: increased knowledge (50% vs. 42%; p = 0.000)^[47]^	NR	NR

CI= confidence interval; IUD=intrauterine device; LARC= Long-Acting Reversible Contraception; p= p value; NR= not reported; OR= Odds ratio; cRCT=cluster randomized control trial; RCT=Randomized control trial; USA=United States of America

‡ Instruments used for risk of bias assessment in primary studies: Blank (2012) utilized criteria developed by National Institute for Health and Clinical Excellene (2009); Jones (2022) utilized Cochrane Collaborative tool for risk of bias in RCTs and the ROBINS-I tool for non-RCT studies; Goueth (2022) utilized criteria developed by the US Preventative Services Task Force

The *informational needs* of young women were: safety concerns, mechanisms of actions, how it affected fertility (related to fear of becoming infertile), protection against sexual transmitted infection, eligibility, and insertion methods (with or without anesthesia) [37,39]. Further, young women expressed the importance of receiving hand-outs of personalized information, for example on side effects, as being complementary to counselling [35]. Specific to LARC, young women overestimated the risk of rare events and were challenged to understand how it worked [37]. Also, women described having a lack of knowledge on availability of contraception, misconceptions about the risk of unprotected sex, and fears about the side effects such as bleeding [38].

The features of contraception option that young women valued were: avoiding intake of hormones, menstrual related effects such as mood swings, and changes in body weight [37]. Furthermore, sexual behaviour related activities young women valued were intercourses as a spontaneous act, and avoiding pregnancy, and sexually transmitted infections [38]. Young women also reported being concerned about losing social standing and image, and not being stigmatized about their sexual activities [34].

Young women’s needs about *support and resources* was described as the influence of the social context in which contraceptive decisions are made such as wider society, religion, peers, parents, community, and partners, influenced values and preferences [37]. Furthermore, it was important for young women to experience privacy in the decision making process and also in option chosen [37]. Friends who had experienced a pregnancy were considered a source of support [38]. Young women wanted access to healthcare services with healthcare professionals providing fact based and a friendly approach that respected confidentiality [34,38]. Some young women experienced financial constraints that influenced their decision about contraception [38].

Personal *characteristics* that influenced their decisions about contraception were: feelings of embarrassment and their religious belief [34,38]. For example, young Muslim women were more likely than orthodox Christian women to favour the use of IUDs [36].

### Effects of interventions to support young women’s contraceptive decisions

Five reviews reported on decision support interventions: two systematic reviews [40,41], one systematic review with meta-analysis [42], one systematic review with narrative synthesis [43], and one scoping review [44]. The evidence on interventions to support decision making for young women considering contraceptive options were primary studies (*n* = 11) reported in reviews rated as moderate [40], low [42], or critically low [41,43,44] according to AMSTAR 2 (see Table 3). According to the JBI critical appraisal items, two reviews offered information for policy [42,43], and two reviews included suggestions on priorities for future research [40,42]. Based on the risk of bias reported in the included reviews, the primary studies were rated as: low risk of bias [45–49], moderate risk of bias [50–52], not reported [53,54] and conflicting results [55]. All the interventions studies were conducted in USA. Detailed information on the interventions is in Table 4.

These reviews reported on two types of decision support interventions: patient decision aids and patient education materials. Four primary studies presented patient decision aids meeting qualifying criteria according to the International Patient Decision Aid Standards presented in the following formats: two websites [45,50], one website with videos and counselling [53], and one computer program [48]. Seven primary studies presented patient education materials in the following formats: one comic in paper format [49], four mobile applications [46,47,52,54] and two waiting room application followed with standard counselling [51,55].

#### Knowledge

Compared to controls, young women exposed to patient decision aids experienced increased knowledge [45,48]. In another study, compared to controls, young women exposed to patient decision aids were more familiar with contraceptive options, however, there was no difference in their knowledge of effectiveness of contraception methods [50]. Patient education material was found to increase knowledge in the intervention group [46,47,49,51,52,54,55]. Only one study reported 43% of participants thought the interventions had too much information [52].

#### Participation in decision making

When young women used a patient decision aid, there was no difference in their level of participation in decision making, compared to controls [45]. One study evaluating patient education material found the intervention group had a higher odds of discussing contraceptive options with their healthcare professional and 70% reported that the intervention aided the quality of the healthcare visit [52].

#### Experiences

Young women who used the patient decision aid had increased confidence in oral contraceptives [48], and had less decisional conflict (not statistical significant) [45]. In one qualitative study, participants described the patient decision aid as informative, narrowing the options, presenting less biased information, and as more useful before the consultation with their healthcare professional [53]. In three studies, patient education materials were positively viewed by participants [49,52], and participants would recommend the patient education material to a friend [51].

## Discussion

Our umbrella review identified 6 reviews describing decisional needs of young women and 5 reviews of interventions to support them making decisions about contraception options. The decisional needs identified were informational needs, young women’s values for features of contraception options, unmet needs for support and resources, and the influence of personal characteristics. Patient decision aids and patient education materials increased knowledge of contraceptive options, and the strength of their effectiveness was supported by primary studies with low risk of bias and reviews with moderate to critically low quality. Two new decisional needs identified by young women considering contraceptive options that were not yet reported in the ODSF were confidentiality and feelings of embarrassment. These needs were reported in other reviews about contraceptive healthcare services [14,56] and they should be considered in the design and delivery of interventions to support young women making these decision. Other decisional needs identified in our review were consistent with those reported by Hoefel et al., in their systematic review of 45 decisional needs assessment studies of adults making healthcare decisions [19]. Both our review and the Hoefel systematic review were based on the ODSF [13].

Lack of knowledge and misconceptions about contraceptive options is of concern given they are decisional needs interfering with achieving quality decisions and common barriers to contraception use [13,14]. Specifically, young women had misconception about insertion and removal of IUDs. Young women globally utilize IUDs to a lesser extent than older women [5]. Recent developments in the field of IUDs [57], may change young women’s attitudes towards IUDs. For example, a recent global survey among gynecologist found that removal is seen as easy, quick procedure which is contrary to some young women who believe removal requires anesthesia [58].

Although younger people tend to be more motivated to be involved in the decision making process, they also tend to lack confidence or even skills to make these decisions and their level of involvement is influenced by health literacy [59]. These personal characteristics were not identified in our review of decisional needs. However, our review of decision support interventions showed that patient education materials targeting young women improved communications about contraception options with their healthcare team and enhanced the quality of their visit [52].

Decision support interventions about contraceptive options identified in our review, patient decision aids and patient education materials, both were more effective than controls for improving knowledge [40–43]. These finding were reported in two reviews with low risk of bias [40,42] and are consistent with a systematic review of 209 patient decision aids [60]. We did not identify studies that evaluated other interventions known to support decision making such as decision coaching or question prompts [15,61,62]. A recent umbrella review investigated the effectiveness of interventions to increase contraceptive use and improve contraception choice among women of all ages [15]. The authors found that motivational interviewing, contraceptive counselling, school-based education, interventions promoting contraceptive access, demand-generation interventions (community and facility based, financial mechanisms and mass media), and mobile phone message interventions increased use of contraceptives [15]. Given that these interventions focused on increasing uptake of contraceptive rather than involving women in choosing the contraceptive option that fits best with their personal circumstances, this study was excluded from our umbrella review. Moreover, the focus on uptake may be an indication that decisional needs may be less prioritized in the research field, and outcomes such as uptake, interest or change in contraceptives are prioritized.

Decision support interventions must be non-directive to facilitate true shared decision making [12]. However, many interventions identified as supporting decision making focus mainly on LARC and were about increasing uptake of LARC options. Guiding young women in which options are most effective is an approach recommended by the WHO [1] and the patient education materials that presented options using a tiered approach did increase uptake of contraceptives [41]. However, there was limited information on these interventions reported in the included studies and it was not clear the extent to which these interventions, included counseling and/or presented balanced, non-directive, information on the options. A recent narrative review aimed to aid clinicians to develop an adolescent-centered, shared decision making approach that respects young women’s choices in regard of their reproductive autonomy [11]. The authors advocated for young women needing information, support, resources, and ways to engage in health care choices independently of their parents or social network. Bearing in mind that social media increasingly influences young women’s healthcare choices [63,64], future research could focus on the distinct decisional needs and digital decision making interventions for young women.

Interestingly, none of the included reviews reported on transgender men or young women with polycystic ovarian syndrome considering contraceptive options. Both groups may benefit from more research on the decision making process of contraceptive options due to the complexity of therapies. For example, transgender men may want to consider fertility preservation rather than permanent contraceptive options [65]. While women diagnosed with polycystic ovarian syndrome, may take medications that could interfere with the effectiveness of contraceptives, due to the altered metabolic function [66].

Our review findings lead to some implications for healthcare professionals and policymakers. Healthcare professionals can facilitate the involvement of young women in these healthcare decisions by recognizing decisional needs as discussed above and providing them with effective interventions such as patient decision aids and patient education materials. However, previous systematic reviews identified barriers to healthcare professionals involving patients in shared decision making and using patient decision aids in clinical practice. Common barriers for healthcare professionals are poor quality information, relational power imbalances, insufficient time, inadequate training, organizational culture that does not support patient involvement, and lack of leadership support [67–69]. Patient decision aids are effective interventions to address the poor-quality information and support patient involvement in decision making [70]. To successfully implement patient decision aids, decision aids should be co-produced with those who are target users such as young women considering contraceptive options, provide training to the whole healthcare team, prepare or invite patients to participate in the decision, get organizational support, and measure use of patient decision aids and patient outcomes as part of a quality improvement process [71]. From a policymaker perspective, patient decision aids are more likely to be used if they are endorsed by governments and healthcare organizations, kept in repositories endorsed by policymakers, and if there are financial incentives to use them [72–74].

### Strengths and limitations

According to the AMSTAR2 the overall quality of evidence is based on reviews scoring low or critical low; only one review scored moderate for interventions to support decision making [40]. Ten of the 11 reviews included where rated critical low or low in accordance with AMSTAR2. This may affect the trustworthiness of the review’s findings and suggest there is a need for future rigorous research focused on young women aged 11–30. Another limitation of our study is the heterogeneity of the risk of bias assessment that we recounted from the primary intervention studies; making it challenging to compare risk of bias assessment findings across studies. Interestingly, the included interventions studies were all conducted in the United States. Due to different healthcare and legal systems globally, young women have different opportunities for access to contraceptives [14]. In most settings young females from lower socioeconomic backgrounds had greater challenges accessing any healthcare services including access to contraceptives [14]. Our findings may be less generalizable to other geographical setting and populations with lower socioeconomic status. Finally, having conducted an umbrella review, our findings were limited to the inclusion criteria of those reviews.

The strength of the current umbrella review is the that two independent reviewers screened both at abstract and full-text level for eligibility, as well as extracted data for the included studies. We conducted a comprehensive search of six databases with our search strategy PRESSed by another information specialist. Another strength is that our knowledge synthesis was theoretically grounded using the ODSF.

## Conclusions

Our umbrella review identified six publications that reported on the decisional needs of young women considering contraceptive options and five publications of decision support interventions aimed at young women. Decisional needs of young women indicated the need for better sources of information on contraceptive options, confidential support from healthcare professionals, financial resources to access a broader range of contraceptive options, and important features of contraceptive options that young women value. Unique decisional needs for young women was having confidential contraceptive services to minimize feelings of embarrassment. Decision support interventions such as patient decision aids and general patient education materials improved knowledge, increased confidence in the chosen contraception option, and resulted in less decisional conflict. Further research should include more interventions aimed at supporting young women’s making contraceptive decisions, and exploring unique decisional needs of young women who have polycystic ovarian syndrome and transgender men.

### Electronic supplementary material

Below is the link to the electronic supplementary material.

Supplementary Material 1

## Data Availability

The datasets used and/or analysed during the current study is available from the corresponding author on reasonable request. Authors have no relevant financial relationships or perceived, potential or real conflicts of interest to disclose.

## Abbreviations

IUD: Intrauterine devices
JBI: Joanna Briggs Institute
LARC: Long-acting reversible contraceptives
ODSF: Ottawa Decision Support Framework
PICOS: Population, Intervention and phenomenon of Interest, Context, Outcomes of Interest, and Study design
PRISMA: Preferred Reporting Items for Systematic Reviews and Meta-Analyses
WHO: World Health Organization
