# Supplementary material for: Decisional needs and interventions for young women considering contraceptive options: an umbrella review
Source: BMC Womens Health. 2024 Jun 8;24:336. doi: 10.1186/s12905-024-03172-2 (PMC11162067; doi:10.1186/s12905-024-03172-2)
Supplement: Supplementary file 1 — Supplementary Material 1 [file 12905_2024_3172_MOESM1_ESM.docx]

**Appendix A: Screening strategy for Ovid MEDLINE(R)**

ALL &lt;1946 to March 03, 2023&gt;

 1        exp Contraception/ or contraceptive agents/ or exp contraceptive agents, female/ or exp contraceptive agents, hormonal/ or contraceptive devices/ or exp contraceptive devices, female/ or Contraception Behavior/        108368

2        (contracept* or &quot;family planning&quot; or &quot;birth control&quot;).tw,kf.        100137

3        ((control* or inhibit* or prevent* or regulat* or suppress*) adj2 (ovulat* or fertili* or pregnan* or conception or reproduct*)).tw,kf.        40764

4        or/1-3        179064

5        Decision Making/ or decision support techniques/ or choice behavior/        154557

6        (decision* adj1 (support* or aid* or making or counsel*)).tw,kf.        213730

7        (choice* or preference*).tw,kf.        554603

8        or/5-7        813975

9        (systematic review or meta-analysis).pt.        303302

10        meta-analysis/ or systematic review/ or systematic reviews as topic/ or meta-analysis as topic/ or &quot;meta analysis (topic)&quot;/ or &quot;systematic review (topic)&quot;/ or exp technology assessment, biomedical/ or network meta-analysis/        340956

11        ((systematic* adj3 (review* or overview*)) or (methodologic* adj3 (review* or overview*))).ti,ab,kf.        305877

12        ((quantitative adj3 (review* or overview* or synthes*)) or (research adj3 (integrati* or overview*))).ti,ab,kf.        15084

13        ((integrative adj3 (review* or overview*)) or (collaborative adj3 (review* or overview*)) or (pool* adj3 analy*)).ti,ab,kf.        37641

14        (data synthes* or data extraction* or data abstraction*).ti,ab,kf.        38866

15        (handsearch* or hand search*).ti,ab,kf.        10948

16        (mantel haenszel or peto or der simonian or dersimonian or fixed effect* or latin square*).ti,ab,kf.        34637

17        (met analy* or metanaly* or technology assessment* or HTA or HTAs or technology overview* or technology appraisal*).ti,ab,kf.        11871

18        (meta regression* or metaregression*).ti,ab,kf.        13975

19        (meta-analy* or metaanaly* or systematic review* or biomedical technology assessment* or bio-medical technology assessment*).mp,hw.        450091

20        (medline or cochrane or pubmed or medlars or embase or cinahl).ti,ab,hw.        328332

21        (cochrane or (health adj2 technology assessment) or evidence report).jw.        21269

22        (comparative adj3 (efficacy or effectiveness)).ti,ab,kf.        17139

23        (outcomes research or relative effectiveness).ti,ab,kf.        11054

24        ((indirect or indirect treatment or mixed-treatment or bayesian) adj3 comparison*).ti,ab,kf.        4242

25        (multi* adj3 treatment adj3 comparison*).ti,ab,kf.        288

26        (mixed adj3 treatment adj3 (meta-analy* or metaanaly*)).ti,ab,kf.        178

27        umbrella review*.ti,ab,kf.        1331

28        (multi* adj2 paramet* adj2 evidence adj2 synthesis).ti,ab,kf.        13

29        (multiparamet* adj2 evidence adj2 synthesis).ti,ab,kf.        18

30        (multi-paramet* adj2 evidence adj2 synthesis).ti,ab,kf.        11

31        ((scoping or rapid or realist) adj3 (review* or overview*)).ti,ab,kf.        22949

32        or/9-31        668616

33        4 and 8 and 32        470

34        limit 33 to yr=&quot;2000 -Current&quot;        438

**Appendix B: List of reasons for exclusion at full-text screening**

| Justification for exclusion | First author of review, year | First author of review or primary study, year |
| --- | --- | --- |
| Not about decision making | Excluded at full-text review level | Ames 2019; Baynes 2022; Botfield 2016; Brittain 2018; Coombe 2016; Courtenay 2022; de Araujo 2015; Deitch 2019; Diaz 2022; Gallo 2008; Goesling 2014; Gormley 2021; Inoue 2015; James-Hawkins 2018; Mahony 2021; Mesfin 2016; Ninsiima 2021; Paine 2000; Ren 2022; Sheikh 2018; Wilkes 2020; Zapata 2015 |
|  | Identified umbrella review | D’Souza 2022: Askari, 2019; Brittain 2015; Edelman 2015; Mooney-Somers, 2019;  D’Souza 2023: Arrowsmith, 2012; Aslam 2017; Carter 2015; DeNicola, 2020; Jawad, 2019; Kirby 2008; Korachais, 2016; Kim 2008; Lopez 2014; Lopez 2016; Mack, 2019; Mason-Jones 2012; Riedel, 2020; Smith 2015; Zapata, 2018; Zulu, 2020; Wakhisi 2011; Wilson 2015 |
|  | Excluded primary studies within reviews | (Blank, 2012 (Hughes, 1995; Wilson, 1994; Baraitser, 2002; Brindis, 2005; Greene, 2005; Kissinger, 1997; Reed, 1999; Hanna, 1993; Edwards, 2008))  (Cavallaro, 2020 (Berenson, 2012; Castano, 2012; Gibbs, 2016; Grubb, 2018; Ferguson, 1998)  (Jones, 2022 (Grubb, 2018; Vayngortin, 2020))  (Walker, 2020 (Martyn, 2006; Martyn, 2013)) |
| Young women is not the focus | Excluded at full-text review level | Alomair 2020; Alspaugh 2020; Ayorinde 2021; Belaid 2016; Burke 2022; Daniele 2017; Danna 2021; Dewart 2019; Edmonds 2014; Gerchow 2022; Hall 2012; LeGuen 2021; Linton 2023; Lopez 2013; Mangone 2016; Moos 2003; Morison 2021; Noone 2002; Parsekar 2021; Pazol 2018; Phiri 2015; Poprzeczny 2020; Prata 2017; Roberts 2022; Rousseau 2019; Silva 2022; Vargas 2019; Wulifan 2016; Wyatt 2014; Yeh 2022; |
|  | Identified umbrella review | D’Souza 2022: Ayanmore, 2016; Ayoola, 2007; Black, 2012;  D’Souza 2023: Aung, 2020; Blacklock, 2016; Bellows Ben, 2016; Sharma, 2018 |
|  |  |  |
|  | Excluded primary studies within reviews | (Baxter, 2011 (Baraitser, 2003; Burack, 2000; Chambers, 2002; Coleman, 2008; Craig, 2006; Croghan, 2006; Donovan, 1997; Donnelly, 2000; French, 2002; French, 2005; French, 2007; Garside, 2002; Griffiths, 2008; Hagley, 2002; Higginbottom, 2006; Ingram, 2007; Jones, 1997; Lester, 2006; Mackereth, 1996; Morrison, 1997; Nwokolo, 2009; Parkes, 2004; Pearson, 2003; Powell, 2008; Reeves, 2006; Ross, 2007; Salmon, 2008; Samangaya, 2007; Sixsmith, 2006; Stanley, 2005; Stone, 2003; Thomas, 2006; van Teijlingen, 2007))  (Daley, 2014 (Aarons, 2002; Horner, 2009; Mendez, 2011; Raine, 2010))  (Fox, 2018 (Becker, 2008; Becker, 2009; Dehlendorf, 2013; Guendelman, 2000; Lowe, 2005; Pilgrim, 2014; Schwarz, 2013; Soleimanpour, 2010; Sonenstein, 1995; Weisman, 2002))  (Kirubarajan, 2022 (Dempsey, 2012; Sharma, 2021))  (Ti, 2022 (Galloway, 2017; Higgins, 2014; Kikalova, 2014; Larkins, 2007; Latka, 2008; Sanders, 2014; Shilo, 2015; van der Geugten, 2017; Whittaker, 2010))  (Cavallaro, 2020 (Dehlendorf, 2017; Garbers, 2012; Garbers, 2012; Kofinas, 2014; Koo, 2017; Sridhar, 2015; Farrokh-Eslamlou, 2014; George, 2015; Leon, 2003; Nawar, 2004; Canto de Cetina 2001; Lei, 1996; Modesto, 2014; Patel, 2003; Demir, 2006; Chin-Quee, 2007; Amataya, 1994; Jain, 2012; Kim, 1992; Leon, 2003; Madden, 2013; Sanogo, 2003; Sapkota, 2017; Wu, 2003; Kim, 2003; Gillespie, 2009; Lee, 2015; Yassin, 2005; El-Khoury, 2016; Terefe, 1993))  (Goueth, 2022 (Akinola, 2019; Chuang, 2019; Garbers, 2012; Garbers, 2012; Wilson, 2014; Koo, 2017; Madden, 2020; Peipert, 2007; Schwarz, 2013;Stephenson, 2020; Sridhar, 2015))  (Walker, 2020 (Dehlendorf, 2017; French, 2014)) |
| Wrong study design | Excluded at full-text review level | Alrawi 2021; Caddy 2023; Chola 2018; Commendador 2010; Crosignani 2012; Dehlendorf 2016; Gonsalves 2016; Halwani 2021; Hoopes 2021; Jianzhong 2014; Kharbouch 2022; Nimbi 2019; Pienkowski 2018; Poprzeczny 2018; Poprzeczny 2019; Sedlecky 2016; Simons 2017; Srikanthan 2008; VanDerPoela 2018; |
|  | Excluded primary studies within reviews | (Walker, 2020 (WHO, 2005; Monasterio, 2010; Rogstad, 2014; Ashby, 2015; Holt, 2017; Faculty of sexual and reproductive health, 2015; Brook, 2014; Radcliffe, 2012; Faculty of sexual and reproductive health, 2010; Rogstad, 2010)) |
| Health conditions (e.g., rheumatoid arthritis, cancer, psychiatric disorders) | Excluded at full-text review level | Ackerman 2016; Bacopoulou 2010; Harris 2022; Horner-Johnson 2019; McCloskey 2020; McCloskey 2021; Peate 2009; Purewal 2018; Rance 2019; Roe 2016; Ross 2022; Skrovanek 2020; Terplan 2015; Tilley 2012; Tong 2015; Verlenden 2019; Weckesser 2013; Yermachenko 2020 |
| Decisional needs for young women living in global south | Excluded at full-text review level | Chola 2023; |
|  | Identified umbrella review | D’Souza 2022: Ackerson, 2019; Blackstone,2017; Huda 2017; James Hawkins, 2016; Munakampe 2018; Pradhan 2015; Tessema 2016; Williamson, 2009; Yakubu 2018 |
|  | Excluded primary studies within reviews | (Kirubarajan, 2022 (Bastos, 2018; Birhane, 2016; Henry, 2021; Hylton-Kong, 2021))  (Reis, 2018 (Avendano, 2016; Fikree, 2017))  (Ti, 2022 (Ahuja, 2019; Alves, 2008; de Bruin, 2017; Nishtar, 2013; van der Straten, 2010; Ziyane, 2006)) |
| Wrong patient population (e.g., males, parents, women undergoing abortion, women utilizing emergency contraception, pregnant women) | Excluded at full-text review level | Agbemenu 2022; Charles 2016; Lavelanet 2022; McLaughlin 2022; Morrison 2023; Napier-Raman 2023; Steyn 2016; Tumchuea 2021; Zemlak 2020 |
|  | Excluded primary studies within reviews | (Baxter, 2011 (Bell, 1999; Folkes, 2001; Free 2002; Ziebland, 1998; Ziebland, 2005))  (Daley, 2014 (Spear, 2004; Wilson, 2011))  (Fox, 2018 (Dehlendorf, 2010; Hickey, 2015; Matulich, 2014; Mollen, 2013; Yee, 2011; Yee, 2015))  (Kirubarajan, 2022 (Rose, 2011; Weston, 2012))  (Ti, 2022 (Corbett, 2006; Rocca, 2007; Rose, 2011; Weston, 2012))  (Blank, 2012 (Belzer, 2005; Ekstrand, 2008; Gold, 2004; Harper, 2005; Adams, 1990; Gilliam, 2004; Omar, 2008; Steven-Simon, 2001)  (Cavallaro, 2020 (Bender, 2004; Davidson, 2015; Schunmann, 2006; Smith, 2015; Whitaker, 2016; Zhu, 2009; Ferreira, 2011; Ferreira, 2015; Lohr, 2018; Langston, 2010; Savelieva, 2003; Abdel-Tawab, 1997; Adanikin, 2013; Bolam, 1998; Gilliam, 2004; Kaewkiattikun, 2017; Vural, 2016; Smith, 2002; Akman, 2010; Proctor, 2006; Fatima, 2018; Hardy, 1998; Ndegwa, 2014; Tomlin, 2017; Torres, 2018; Lemani, 2017))  (Goueth, 2022 (Stephenson, 2020)  (Jones, 2022 (Davidson, 2015; Garbers, 2015; Kofinas, 2014; Shafii, 2019)) |
| Not contraception | Excluded at full-text review level | Haakenstad 2022; Johnson 2021; Kraft 2014; Mandal 2017; Norris 2022; Spencer 2014; Templeton 2017; Woldearegay 2021 |
| Older version of an included review | Excluded at full-text review level | Gallo 2005; Lopez 2008; Lopez 2009; Lopez 2013; Pazol 2015; |
| School-based curriculum programs; | Excluded at full-text review level | Blank 2010; Lopez 2016 |
|  | Identified umbrella review | D’Souza 2023: Desrosiers 2020; Tolli 2012 |
| Wrong intervention | Excluded at full-text review level | Lopez 2016 |
|  | Identified umbrella review | D’Souza 2023: Karra, 2016; Khan, 2016 |
| Provider perspective (health care professional) | Excluded at full-text review level | Monteiro 2020; Onukwugha 2019 |
|  | Excluded primary studies within reviews | (Baxter, 2011 (Allen, 2004; Barrett, 2000; Bissell, 2003; Bissell, 2006; Bloxham, 1997; Fairhurst, 2004; Fallon, 2003; French, 2006; Garside, 2000; Hoggart, 2006; Jolley, 2001; Mackie, 2002; Mason, 2005; McCann, 2008; Pitts, 1996; Wellings, 2007; Ziebland, 1998))  (Walker, 2020 (Kim, 2005; Johnson, 2010)) |
| HIV focus | Excluded at full-text review level | Leyva-Moral 2018; Lopez 2013 |
| Language other than english | Excluded at full-text review level | Peon-Ponce 2021 (spanish); Pereira dos Santos 2015 (portuguese) |
| Include only primary studies prior to 2000; | Excluded at full-text review level | Hulton 2001 |
| Condom use | Excluded primary studies within reviews | (Blank, 2012 (Jernott, 2005; Orr, 1996; Smith, 1997; Thompson, 2001; Ullman, 1996;)) |

**Appendix C. Figure 1. Interrelation between reviews and individual studies; decisional needs (made with SankeyMATIC)**

**Appendix D. Figure 2. Interrelation between reviews and individual studies; interventions to support decision making (made with SankeyMATIC)**

**Appendix E: Two items from JBI Critical Appraisal Checklist for Systematic Reviews and Research Syntheses**

| **First author of review (year)** | **JBI 10**  Policy and practice recommendations | **JBI 11**  Directions for new research |
| --- | --- | --- |
| **Decisional needs** | | |
| Baxter (2011) | Unclear | Yes |
| Daley (2014) | Unclear | Unclear |
| Fox (2018) | Unclear | Yes |
| Kirubarajan (2022) | Yes | Yes |
| Reis (2018) | Unclear | Unclear |
| Ti (2022) | Yes | Yes |
| **Decision support interventions** | | |
| Blank (2012) | Yes | Unclear |
| Cavallaro (2020) | Unclear | Unclear |
| Goueth (2022) | Yes | Yes |
| Jones (2022) | Unclear | Yes |
| Walker (2020) | Unclear | Unclear |
